# Nap duration and its association with hypertension-diabetes comorbidity in minority populations: evidence from the CMEC study

**DOI:** 10.3389/fendo.2025.1563944

**Published:** 2025-04-28

**Authors:** Renhua Zhang, Enhui Zhou, Leilei Liu, Yuan Wang, Fei Xiao, Feng Hong

**Affiliations:** School of Public Health, The Key Laboratory of Environmental Pollution Monitoring and Disease Control, Ministry of Education, Guizhou Medical University, Guiyang, China

**Keywords:** hypertension-diabetes comorbidity, minority populations, China Multi-Ethnic Cohort, chronic disease, nap duration

## Abstract

**Objective:**

Limited information is available on the effect of nap duration and hypertension-diabetes comorbidity (HDC) in minority people. We aimed to explore the relationship between nap duration and HDC for the co-management of hypertension and diabetes mellitus in the minority.

**Methods:**

A total of 16,911 participants from the China Multi-Ethnic Cohort (CMEC) were enrolled in this cross-sectional study. Nap duration was then categorized into four groups: 0 hours (reference group), 0–0.5 hours, 0.5–1 hour, and >1 hour. Multiple logistic regression was applied to analyze the association between nap duration and HDC. Restricted cubic splines (RCS) analysis was conducted to assess the nonlinear relationship between nap duration and the co-occurrence of HDC. Subgroup analyses were subsequently performed, stratified by sex, age, and ethnicity.

**Results:**

Among 16,911 participants with a median age of 51.79 years, of whom 66.00% were female. A total of 647 subjects were in the HDC group, representing a prevalence rate of 3.83% in the entire study population. Multivariate logistic regression analysis showed that, after multivariate adjustments, the odds ratios (95% CI) for HDC across the four groups (0h, 0–0.5h, 0.5–1h and > 1h) were: reference, 1.305 (1.027, 1.650), 1.254 (1.016, 1.542), 1.612 (1.261, 2.046), respectively. RCS analyses revealed distinct associations between naptime duration and HDC: no significant relationship in participants aged <45 years (*P-*overall=0.529); a linear positive correlation in those aged 45–60 years (*P-*overall=0.001); and an inverse J-shaped association peaking at 60 minutes in individuals aged >60 years (*P-*overall=0.026, *P-*nonlinearity=0.015). The subgroup analysis revealed that among >45 years, male, Dong or Miao, a longer nap duration was also associated with an increased prevalence risk of HDC.

**Conclusion:**

Longer napping duration were associated with an increased risk of HDC and monitoring nap duration may aid in identifying high-risk groups.

## Introduction

Napping, or the practice of taking a short daytime sleep, has become an integral part of daily life, particularly in many Asian countries, including China. The prevalence of napping among Chinese adults increases with age, with 61.7% of males and 46.8% of females aged 60 and above reporting a habitual napping behavior (1). Napping is often regarded as a healthy lifestyle practice that contributes to improved mental well-being (2).However, as lifestyles modernize, the duration of napping have become more varied, with some individuals napping for longer durations (3). Emerging research suggests that napping may influence the development of chronic diseases. While short naps are generally associated with improved cognitive function, mental health, and cardiovascular outcomes (4–6), prolonged or irregular napping patterns are linked to a higher risk of developing chronic conditions such as hypertension and diabetes (7, 8). Disrupted sleep patterns, which include excessive daytime sleep, may interfere with circadian rhythms and lead to metabolic disturbances (9).

Hypertension and diabetes are major non-communicable diseases and significant global public health challenges (10). Although they are separate conditions, they frequently coexist, and patients with comorbid hypertension and diabetes face a heightened risk of cardiovascular disease (CVD) and cognitive impairment (CI) that exceeds the sum of the risks associated with either condition alone (11, 12). According to a meta-analysis, the prevalence of hypertension-diabetes comorbidity (HDC) in China has shown an upward trend, reaching 8.7% among adults (13). Notably, racial disparities in the prevalence of multimorbidity may stem from distinct cultural practices and lifestyle habits among ethnic minorities (14). In China’s northwestern regions, the prevalence of HDC among adults is 4.58% (15), while in the eastern coastal regions, among community-dwelling elderly individuals aged 65 and older, the comorbidity rate is 12.5% (16). However, studies examining the prevalence and associated factors of HDC in socioeconomically disadvantaged regions remain limited. Furthermore, ethnic minorities such as the Dong, Miao, and Bouyei in southwestern China face even greater challenges in managing hypertension and diabetes. Compared to urban patients, these ethnic minorities, predominantly residing in rural areas, encounter significant barriers to accessing local health services due to high travel costs and limited healthcare availability (17).

To date, in China, particularly in the southwestern region, no large-scale studies have specifically investigated the association between nap duration and HDC. Instead, most research has primarily focused on individual diseases. It is essential to explore how nap duration could influence the development and progression of co-existence diseases. This study aims to investigate the relationship between nap duration and hypertension-diabetes comorbidity, using epidemiological methods to examine how nap duration may affect metabolic processes and contribute to the risk of these diseases. By focusing on the metabolic health impacts of napping, this research seeks to provide new insights into the prevention and management of hypertension-diabetes comorbidity.

## Methods

### Study design and participants

We conducted a population-based prospective study among three main ethnic minorities in the southwest China, including the Bouyei, Dong, and Miao, aged 30 to 79 years. The data used in this study were derived from the baseline survey conducted in the China Multi-Ethnic Cohort Study from July 2018 to August 2019. Detailed information about the study population has been previously described (18).

**Figure 1:**
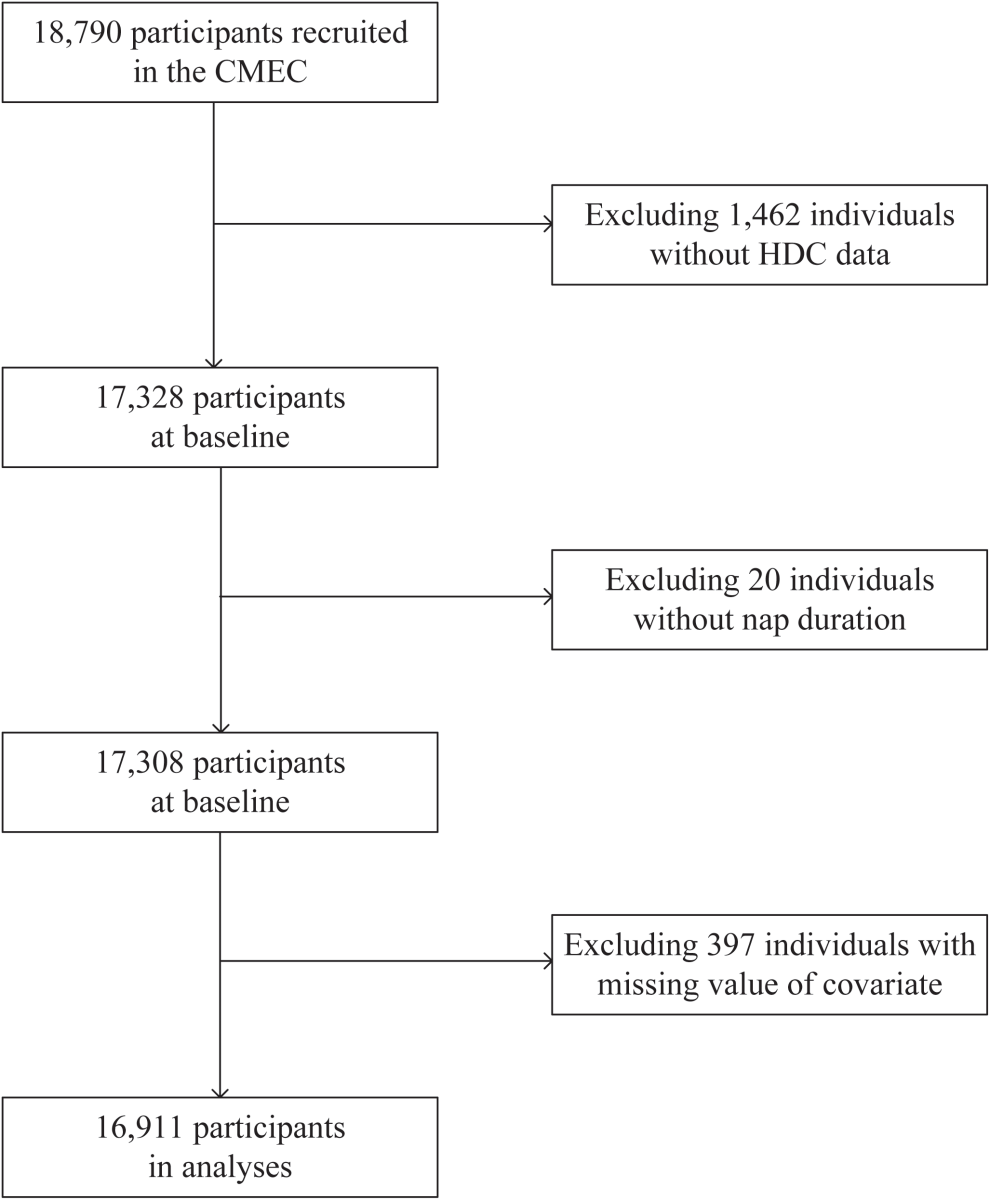
Flowchart of study participants.

Exclusion criteria: 1) participants without basic information; 2) participants who did not self-report hypertension or diabetes and did not undergo laboratory testing; 3) incomplete data on key variables such as nap duration. **Figure 1** illustrates the details of the participant enrollment process. Finally, a total of 16,911 individuals were included in this analysis. Each participant completed electronic questionnaires through face-to-face interviews and underwent physical examinations and clinical laboratory tests. This study was approved by the Medical Ethics Review Committee of Sichuan University (K2016038) and the Research Ethics Committee of The Affiliated Hospital of Guizhou Medical University (2018[094]), and all methods were performed in accordance with relevant guidelines and regulations. Written informed consent was obtained from each participant before study initiation.

### Exposure – self-reported duration of daytime napping

Participants were first asked whether they had a habit of napping. If they answered yes, they were further asked: “How long, on average (in minutes), do you usually nap after lunch?” Based on their responses to these two questions, participants were categorized into four groups: no daytime napping (0 min/day), short daytime napping (1–30 min/day), moderate daytime napping (31–60 min/day), and long daytime napping (>60 min/day) (19).

### Outcome – HDC

In this study, diabetes was defined as meeting any of the following criteria: (1) self-reported physician-diagnosed diabetes, (2) fasting plasma glucose (FPG) ≥ 7.0 mmol/L or glycosylated hemoglobin (HbA1c) ≥ 6.5%. Hypertension was defined as: (1) self-reported physician-diagnosed hypertension, or (2) systolic blood pressure (SBP) ≥ 140 mmHg or diastolic blood pressure (DBP) ≥ 90 mmHg. HDC was defined as the coexistence of both hypertension and diabetes.

### Covariates

Face-to-face structured questionnaires were used to collect demographic information and lifestyle factors, including demographics (sex, age, and ethnicity), socioeconomic gradient (residence and annual household income), health behaviors (smoking (20), alcohol consumption (21), physical activity, duration of night sleep, sleep disorder), central obesity and salt intake per day. Physical activity considered participants’ occupational, traffic, chores, and leisure time activities, and were divided into low and high based on the median value of metabolic equivalent for task (MET).Night sleep duration and sleep disorders were classified into three types of insomnia based on participants’ responses to the following questions: (1) Disorders of Initiation and Maintenance (DIMS): defined as taking more than 30 minutes to fall asleep at least three days per week; (2) Early Morning Awakening (EMA): defined as waking up early in the morning and being unable to fall back asleep at least three days per week; (3) Daytime Dysfunction (DDF): defined as difficulty staying awake during the day while working, eating, or talking to others at least three days per week. The total number of insomnia types reported by each participant was summed and categorized as follows: 0 (none), 1 (any one type), 2 (any two types), and 3 (all three types) (22).

### Statistical analysis

The baseline demographic characteristics of the participants were described according to the four levels of nap duration (no nap, ≤30 min, 31–60 min, and >60 min). The Kolmogorov–Smirnov (K–S) test was used to assess the normality of continuous variables. Normally distributed continuous variables were presented as mean ± standard deviation (SD), while non-normally distributed variables were summarized as mean (SD) and median (25th, 75th percentiles). Categorical variables were expressed as frequency counts and percentages. Group comparisons for continuous variables were performed using the Kruskal–Wallis H test, and the Chi-square test was used for categorical variables. Restricted cubic spline (RCS) analysis was employed to explore the nonlinear relationship between nap duration and the risk of hypertension-diabetes comorbidity (HDC). Multiple logistic regression analyses were used to evaluate the associations between nap duration and HDC. Model 1 was an unadjusted crude model. In Model 2, we adjusted for sex, age, ethnicity. Model 3 further adjusted for central obesity, smoking status, alcohol consumption, annual household income, physical activity, salt intake, night sleep duration, and night sleep disorders based on Model 2. In addition, in different age groups (<45 years, 45–60 years, and >60 years), restrictive cubic splines (RCS) were used to describe the potential relationship between nap duration and HDC risk (23). Subgroup analyses were conducted to explore the potential modifiers of the association between nap duration and HDC, stratified by sex, age (<45 years, 45–60 years, and >60 years), and ethnicity (Dong, Miao, and Bouyei). All statistical analyses were performed using R software (version 4.3.1). A two-sided P-value < 0.05 was considered statistically significant.

## Results

### Population characteristics

**Figure 2:**
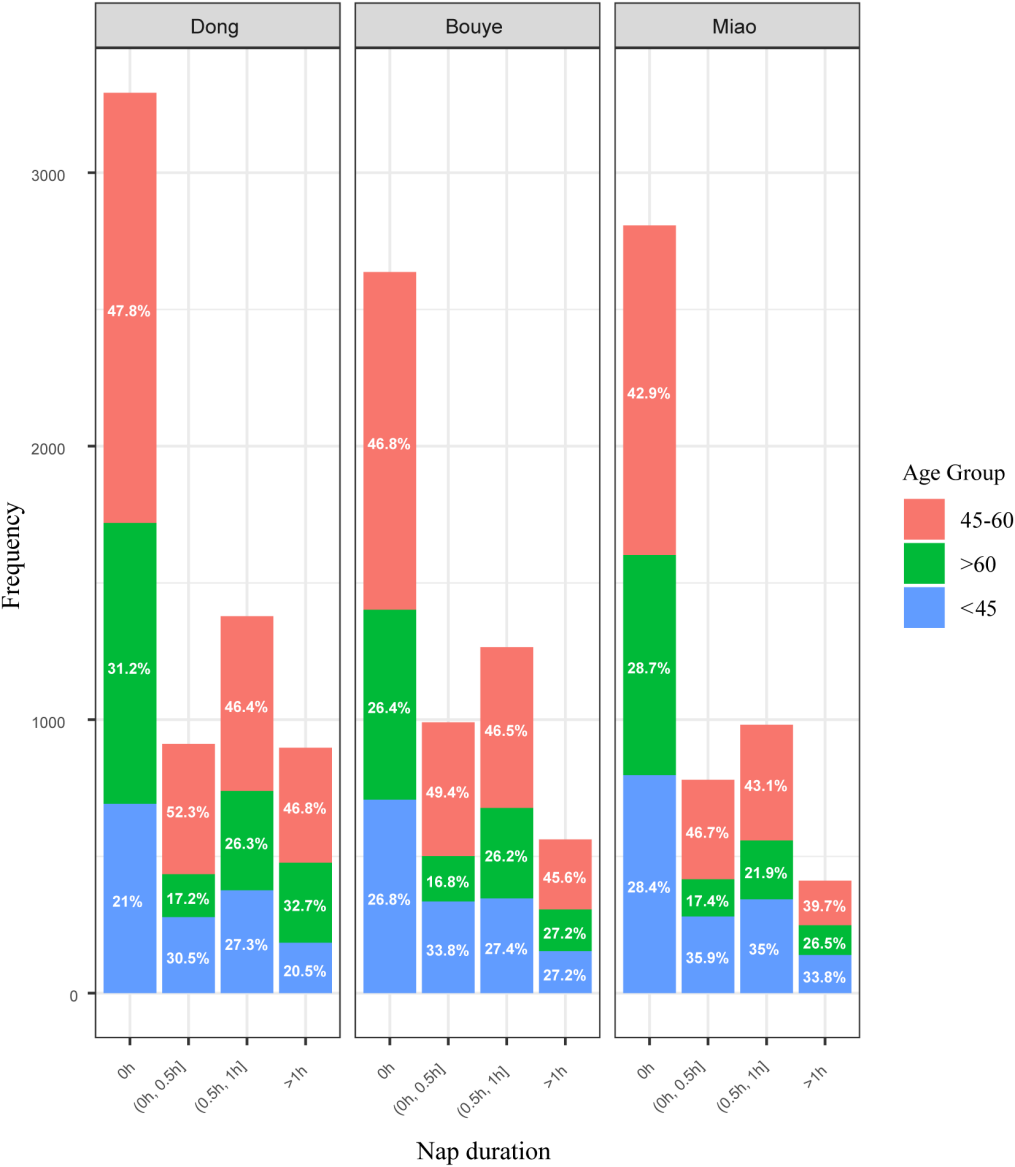
Distribution of different ethnic groups and age groups across various nap duration categories.

The data presented in **Figure 2** indicates “0 h” is the most common nap duration, with proportions decreasing as nap time increases across all groups. The 45–60 age group dominates most categories, particularly in longer nap durations, while the <45 group shows a higher proportion in shorter naps. Variations in nap patterns reflect differences across ethnicities and age groups.

The study, which involved 16,911 participants classified by nap duration into groups of no nap (0h), naps between 0 and 0.5 hours, 0.5 to 1 hour, and greater than 1 hour, revealed several key trends. The detailed data are presented in **Table 1**. Regarding age and sex, participants with longer nap durations were generally older, and the proportion of males was higher in these groups compared to those with no or shorter naps. In terms of ethnicity and residence, ethnic distribution varied across nap - duration groups, with the Dong ethnic group more common in longer - nap groups, and participants from rural areas were more likely to have no nap or longer naps. Considering socio - economic factors, individuals with lower household incomes were more prone to report longer nap durations, especially the lowest - income group in the no - nap and >1h nap categories. For lifestyle factors, longer nap durations were associated with lower physical activity levels, increased alcohol consumption, higher salt intake, and a greater likelihood of sleep disorders. Finally, in relation to health outcomes, the prevalence of HDC was higher among those with longer nap durations, suggesting a potential link between longer naps and an increased risk of chronic disease.

**Table 1:** Characteristics of the qualified study participants.

	Overall	0h	(0h, 0.5h]	(0.5h, 1h]	>1h	*P* value
N	16911	8736	2681	3624	1870	
Age, median (IQR)	51.79 [44.27, 60.66]	52.67 [44.96, 61.86]	49.28 [43.00, 55.77]	51.01 [43.71, 60.10]	52.49 [44.81, 61.82]	<0.001
Sex, n (%)						<0.001
Male	5750 (34.00)	2585 (29.59)	937 (34.95)	1437 (39.65)	791 (42.30)	
Female	11161 (66.00)	6151 (70.41)	1744 (65.05)	2187 (60.35)	1079 (57.70)	
Ethnicity, n (%)						<0.001
Dong	6478 (38.31)	3292 (37.68)	911 (33.98)	1378 (38.02)	897 (47.97)	
Bouyei	5454 (32.25)	2637 (30.19)	990 (36.93)	1265 (34.91)	562 (30.05)	
Miao	4979 (29.44)	2807 (32.13)	780 (29.09)	981 (27.07)	411 (21.98)	
Residence, n (%)						<0.001
Rural	13381 (79.13)	7394 (84.64)	1779 (66.36)	2632 (72.63)	1576 (84.28)	
Urban	3530 (20.87)	1342 (15.36)	902 (33.64)	992 (27.37)	294 (15.72)	
Annual household income, n (%)						<0.001
<100000 CNY	15440 (91.30)	8176 (93.59)	2278 (84.97)	3218 (88.80)	1768 (94.55)	
100000-199999 CNY	1315 ( 7.78)	497 ( 5.69)	365 (13.61)	364 (10.04)	89 ( 4.76)	
≥200000 CNY	156 ( 0.92)	63 ( 0.72)	38 ( 1.42)	42 ( 1.16)	13 ( 0.70)	
Central obesity, n (%)^a^						0.584
No	5538 (32.75)	2868 (32.83)	899 (33.53)	1180 (32.56)	591 (31.60)	
Yes	11373 (67.25)	5868 (67.17)	1782 (66.47)	2444 (67.44)	1279 (68.40)	
Smoke, n (%)						<0.001
Never	13531 (80.01)	7225 (82.70)	2214 (82.58)	2755 (76.02)	1337 (71.50)	
Current	2754 (16.29)	1240 (14.19)	371 (13.84)	707 (19.51)	436 (23.32)	
Ever	626 ( 3.70)	271 ( 3.10)	96 ( 3.58)	162 ( 4.47)	97 ( 5.19)	
Alcohol, n (%)						<0.001
Never	8627 (51.01)	4804 (54.99)	1232 (45.95)	1711 (47.21)	880 (47.06)	
Occasionally	6256 (36.99)	3030 (34.68)	1153 (43.01)	1414 (39.02)	659 (35.24)	
Frequently	2028 (11.99)	902 (10.33)	296 (11.04)	499 (13.77)	331 (17.70)	
Salt intake, median (IQR)	49.71 [34.52, 69.04]	51.40 [34.52, 69.04]	48.33 [34.25, 68.08]	49.32 [34.52, 69.04]	53.70 [38.07, 76.32]	<0.001
Physical activity, n (%)^b^						<0.001
Low	5598 (33.10)	2765 (31.65)	897 (33.46)	1252 (34.55)	684 (36.58)	
Moderate	5659 (33.46)	2861 (32.75)	928 (34.61)	1251 (34.52)	619 (33.10)	
High	5654 (33.43)	3110 (35.60)	856 (31.93)	1121 (30.93)	567 (30.32)	
Sleep duration, median (IQR)	7.00 [6.00, 8.00]	7.00 [6.00, 8.00]	7.00 [6.00, 8.00]	7.00 [6.00, 8.00]	7.00 [6.00, 8.00]	<0.001
Sleep disorder, n (%)						<0.001
0	9047 (53.50)	4553 (52.12)	1497 (55.84)	1999 (55.16)	998 (53.37)	
1	3326 (19.67)	1747 (20.00)	467 (17.42)	728 (20.09)	384 (20.53)	
2	2977 (17.60)	1575 (18.03)	469 (17.49)	585 (16.14)	348 (18.61)	
3	1561 ( 9.23)	861 ( 9.86)	248 ( 9.25)	312 ( 8.61)	140 ( 7.49)	
HDC, n (%)						<0.001
No	16264 (96.17)	8459 (96.83)	2570 (95.86)	3468 (95.70)	1767 (94.49)	
Yes	647 ( 3.83)	277 ( 3.17)	111 ( 4.14)	156 ( 4.30)	103 ( 5.51)	

CNY, Chinese Yuan Renminbi; h, hour(s); IQR, interquartile range.

a Defined by tertiles of the hours of MET per day: low (MET < 16.7), moderate (16.7 ≤ MET < 32.4), and high (MET ≥ 32.4).

b Defined as a waist-to-hip ratio of 0.85 or higher for women and 0.9 or higher for men.

### Observational analyses of nap duration on HDC

The results of nap duration and HDC in Multiple logistic regression are shown in **Table 2**. In the crude model, long nap duration(>1h) (OR = 1.768, 95% CI: 1.402, 2.214; *P* < 0.001) had significantly higher risk of HDC compared to those who slept in daytime at all. The positive association of nap duration and HDC was still statistically significant though attenuated after adjusting for age, sex, ethnicity, residence, annual household income, central obesity, smoke, drink, salt intake, physical activity, night sleep duration, night sleep disorder (OR = 1.638, 95% CI: 1.293, 2.061; *P* < 0.001). Further adjustment of chronotype and snoring did not change the positive association between nap duration group and HDC (OR = 1.612, 95% CI: 1.261, 2.046; *P* < 0.001).

**Table 2:** Association of nap duration and HDC in Multivariable logistic regression.

Nap duration	N	Model 1^a^		Model 2^b^		Model 3^c^	
		*OR* (95% CI)	*P* value	*OR* (95% CI)	*P* value	*OR* (95% CI)	*P* value
0h	9756	Ref		Ref		Ref	
(0h, 0.5h]	2962	1.318 (1.052, 1.639)	0.015	1.553 (1.235, 1.941)	<0.001	1.305 (1.027, 1.650)	0.027
(0.5h, 1h]	3960	1.361 (1.114, 1.655)	0.002	1.386 (1.132, 1.691)	0.001	1.254 (1.016, 1.542)	0.033
>1h	2063	1.768 (1.402, 2.214)	<0.001	1.638 (1.293, 2.061)	<0.001	1.612 (1.261, 2.046)	<0.001

a Covariables in model 1: no confounding factors were adjusted.

b Covariables in model 2: age, sex, ethnicity,

c Covariables in model 3: Model 2 + residence, annual household income, central obesity, smoke, drink, salt intake, physical activity, night sleep duration, night sleep disorder. OR indicates odds ratio; Ref, reference; CI, confidence interval.

### Dose–response relationship between nap duration and HDC in different age group

**Figure 3:**
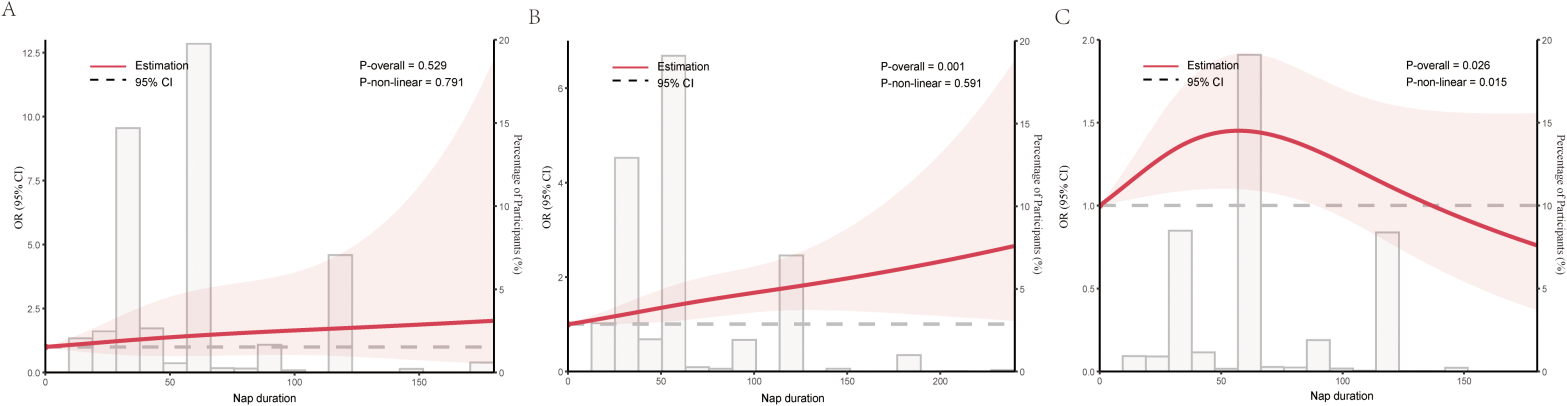
Multivariable restricted cubic spline of nap duration. **(A)** <45 years; **(B)** 45–60 years; **(C)** >60 years. Solid lines indicate ORs, and shadow shape indicate 95% CIs. The gray column depicts the percentage of participants of different durations of napping.

Participants were stratified into three age groups: 30–45, 45–60, and 60–80 years (20). In the group aged <45 years, no significant nonlinear association was observed between naptime duration and HDC (*P* for overall = 0.529; *P* for nonlinearity = 0.791) (**Figure 3A**). For participants aged 45–60 years, HDC probability increased linearly with longer naptime duration (*P* for overall = 0.001; *P* for nonlinearity = 0.591) (**Figure 3B**). In contrast, an inverse J-shaped correlation (peaking at 60 minutes) was identified in those aged >60 years (*P* for overall = 0.026; *P* for nonlinearity = 0.015) (**Figure 3C**).

### Subgroup analysis

**Figure 4:**
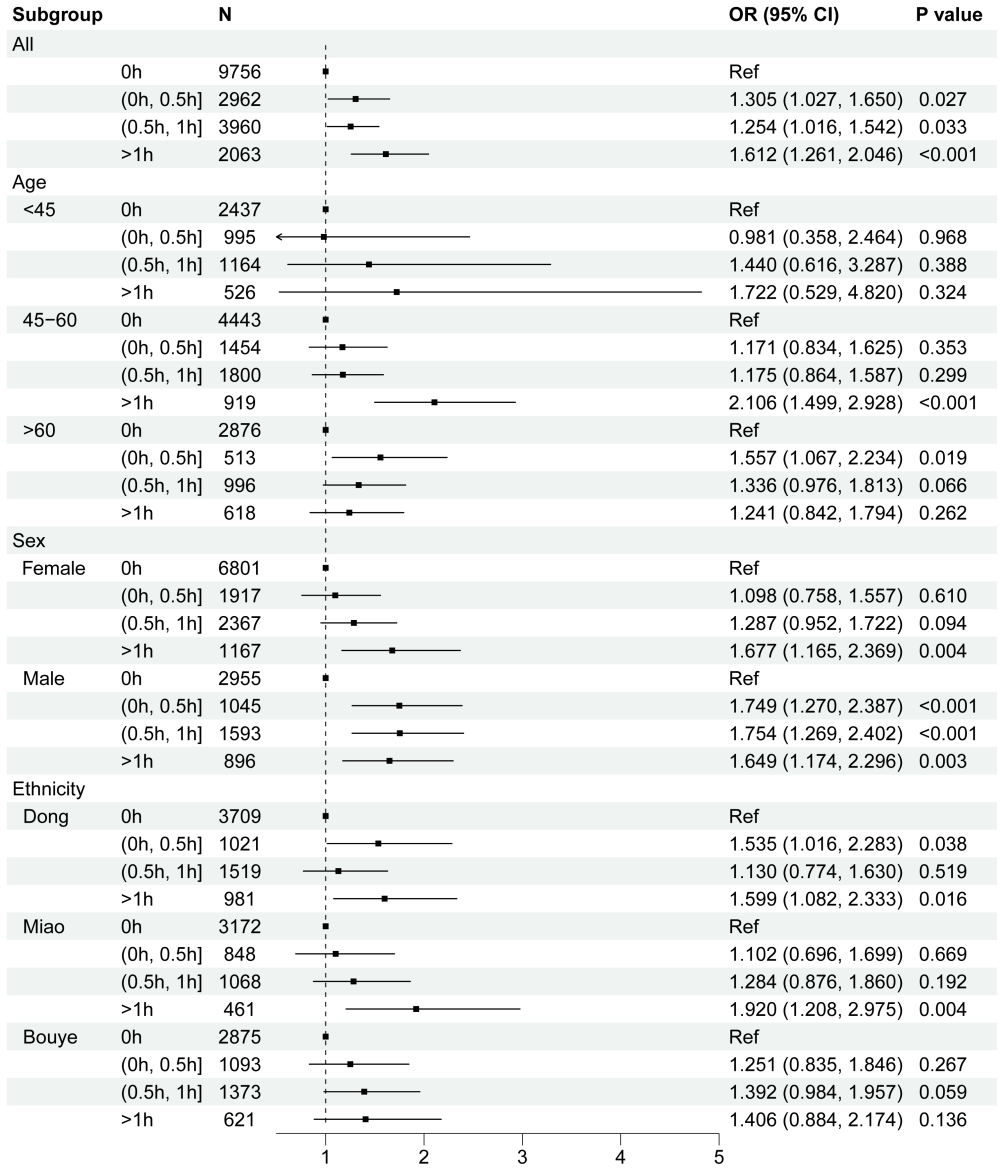
Forest plot for stratified analyses of the associations between nap duration and the risk of HDC for age, sex, ethnicity, residence, annual household income, central obesity, smoke, drink, salt intake, physical activity, sleep duration, sleep disorder. Each group were adjusted for the other covariates except itself.

The subgroup analysis of nap duration and risk of HDC stratified by age, sex, and ethnicity are conducted to investigate whether there had differences among groups. The results are presented in **Figure 4** (forest plot) and **Supplementary Tables S1–S3**.

The subgroup analysis examining the association between nap duration and HDC, stratified by age, sex, and ethnicity, identified several notable findings. Among younger individuals, both short naps (0.5–1 h) and prolonged naps (>1 h) were associated with an increased risk of HDC compared to non-nappers. However, these associations were not statistically significant in the fully adjusted model (0.5–1 h: OR = 1.440, 95% CI: 0.616–3.287, *P* = 0.388; >1 h: OR = 1.722, 95% CI: 0.529–4.820, *P* = 0.324). For individuals aged 46–60, naps lasting more than 1 hour were significantly associated with a higher risk of HDC compared to non-nappers (model 3: OR = 2.106, 95% CI: 1.499–2.928, *P* < 0.001). Among older adults (>60 years), short naps (0–0.5 h) also showed an increased risk of 55.7% (OR = 1.557, 95% CI: 1.067–2.234), while no significant association was observed for naps lasting 0.5–1 h (OR = 1.336, 95% CI: 0.976–1.183). Naps shorter than 30 minutes were linked to an elevated risk of HDC in the fully adjusted model (OR = 1.557, 95% CI: 1.067–2.234, *P* = 0.019).

In the sex-stratified analysis, females with nap durations exceeding 1 hour exhibited a significant positive association with HDC (model 3: OR = 1.677, 95% CI: 1.165–2.369, *P* = 0.004). Similarly, in males, prolonged naps (>1 h) were consistently associated with a higher risk of HDC across all models (model 3: OR = 1.649, 95% CI: 1.174–2.296, *P* = 0.003). Ethnicity-stratified analysis revealed that among the Dong ethnic group, both short and long naps were linked to an elevated risk of HDC. For the Miao group, extended naps (>1 h) were associated with an increased risk of HDC compared to non-nappers, even after adjusting for potential confounders. In contrast, no significant associations were observed in the Bouyei group, regardless of nap duration.

## Discussion

The present study explored the association between nap duration and HDC, revealing a significant relationship between nap duration and HDC risk. Our dose-response analysis indicated a clear duration-effect relationship: as nap duration increased, it was associated with an increased risk of HDC. This finding aligns with previous studies, suggesting a potential link between nap duration and the occurrence of hypertension and diabetes.

However, it is important to note that the underlying mechanisms of this association remain unclear. Prolonged napping may reduce energy expenditure and lead to obesity, which plays a significant role in the development of both diabetes and hypertension (24, 25). Additionally, long naps may disrupt individuals’ circadian rhythms of sleep and wakefulness (26, 27). The circadian rhythm, an internal biological clock, governs a wide array of physiological processes in the body. When long naps occur, they can misalign this internal clock. This misalignment directly affects the timing of hormone release, including insulin (28). With circadian rhythm disruption due to long naps, the normal pattern of insulin secretion is disrupted. For instance, insulin may be released at inappropriate times or in insufficient amounts (29). This disruption in insulin secretion leads to reduced insulin sensitivity. Insulin sensitivity refers to the body’s cells’ ability to respond effectively to insulin. When sensitivity decreases, cells do not take up glucose from the bloodstream as efficiently (30). As a result, blood glucose levels rise, and over time, this can lead to insulin resistance. Insulin resistance is a key precursor to both diabetes and hypertension (31). In relation to hypertension, insulin resistance can trigger a cascade of physiological changes. It can lead to increased sodium reabsorption in the kidneys, which in turn increases blood volume and ultimately raises blood pressure (32). All these factors ultimately contribute to HDC (33).

Although previous studies have highlighted the positive effects of daytime napping on learning, memory, work performance, and energy restoration (34, 35), most research has focused on the general population. This study, however, targeted ethnic minorities in southwest China, particularly the Dong, Miao, and Bouye groups. These groups have distinct cultural backgrounds, and socioeconomic conditions compared to the Han population (36, 37), which may uniquely influence the relationship between nap duration and HDC. For example, research has shown a positive correlation between higher socioeconomic status and hypertension prevalence among individuals with self-reported diabetes (38). In this study, among those with nap durations >1 hour, 47.55% were from the Dong ethnic group, with a higher proportion of individuals aged >60 years. In contrast, these proportions were only 30.10% and 22.35% among the Bouyei and Miao ethnic groups, respectively. This may explain why the relationship between nap duration and HDC differs across ethnic groups, as ethnicity could account for these discrepancies. Our study fills this research gap.

Moreover, our findings highlighted that individual who napped for more than 1 hour, particularly those aged 45–60 years from minority groups, had a significantly higher risk of HDC. While existing studies on napping have primarily focused on individuals aged 45 years and older, we categorized adults into three groups (<45, 45–60, and >60 years), thereby expanding the scope of research. Notably, we observed that the risk of HDC significantly increased only in the 45–60 age group when nap duration exceeded 1 hour compared to those who did not nap, which is consistent with previous findings (39).For individuals aged 45–60 years, prolonged nap durations likely reflect excessive fatigue or poor lifestyle habits, such as irregular sleep patterns (40). Extended periods of inactivity, such as naps lasting more than one hour, can adversely affect circulation and metabolism. Prolonged sedentary behavior is closely associated with the increased risk of various chronic conditions, particularly hypertension and diabetes (41, 42), and research has shown that prolonged sitting and physical inactivity can contribute to increased insulin resistance (41), leading to elevated blood glucose levels and negatively impacting cardiovascular health. This finding suggests that regulating nap duration may play a crucial role in the prevention of diabetes and its associated complications.

For individuals aged over 60, however, a nap duration of 0–30 minutes significantly increased the prevalence of HDC. The reduced physiological resilience of older adults means that short naps may be insufficient to provide adequate rest and recovery, particularly when compounded by pre-existing fatigue or physical weakness. A shorter nap duration may reflect an incomplete recovery process, exacerbating chronic fatigue and affecting the endocrine and metabolic systems, which in turn increases the risk of hypertension and diabetes (43, 44). These underlying mechanisms require further investigation to better understand the relationship between nap duration and the comorbidity of hypertension and diabetes.

These results highlight the age-dependent differences in the health impacts of nap duration, likely linked to factors such as physiological needs, lifestyle habits, and the body’s capacity for recovery. Therefore, tailored nap duration recommendations and increased physical activity may help reduce the risk of hypertension and diabetes comorbidity in different age groups. At the level of health policy, research findings indicate a need to strengthen health management of nap habits, particularly by implementing health education and intervention measures in high-risk populations. Governments and health organizations can incorporate reasonable napping into chronic disease prevention and control guidelines, advocate for moderate napping, and avoid the potential health risks of excessive daytime sleep. Additionally, combining individualized health management plans can optimize workplace and community health promotion strategies to reduce the incidence of HDC. In clinical practice, physicians should consider patients’ nap habits when assessing their risk of HDC, as part of a comprehensive evaluation of lifestyle factors. For individuals with a habit of long naps, healthcare professionals can offer appropriate lifestyle adjustment recommendations, such as limiting nap duration, improving nighttime sleep quality (45), and increasing physical activity (46), to reduce the risk of HDC. Furthermore, future research could consider incorporating nap behavior into comprehensive risk assessment models for HDC to enhance the precision of disease prediction and early intervention.

This study utilized a representative large sample database to investigate the relationship between nap duration and the co-occurrence of HDC in ethnic minority populations. To ensure the comprehensiveness and reliability of the results, subgroup analyses were conducted to validate the stability and generalizability of the findings across multiple dimensions. However, several limitations exist in this study. First, the assessment of nap duration was based on subjective self-reported data, which may introduce recall bias and, consequently, affect the accuracy of the data. Second, while the main focus of this study was on nap duration, factors such as nap frequency and timing, which are also important, were not explored. Most importantly, as a cross-sectional analysis, our study design inherently precludes causal inferences between napping and health outcomes. Although this investigation is embedded within an ongoing cohort study with planned longitudinal follow-ups, we strongly agree that future research should employ causal inference approaches—such as longitudinal designs tracking nap patterns over time, or Mendelian randomization studies leveraging genetic variants as instrumental variables—to formally test potential causal relationships (47).

## Conclusion

This study suggests that nap duration is significantly associated with the co-occurrence of HDC in minority populations, with both short and long naps duration increasing the risk of HDC. These findings highlight the complex relationship between rest patterns and metabolic health, emphasizing the need for careful consideration of nap duration as part of health management strategies. Future research should explore the mechanisms behind these associations and whether regulating nap time could be an effective preventive measure, particularly in high-risk ethnic groups.

## Data Availability

The data that support the findings of this study are available on request from the corresponding author. Requests to access the datasets should be directed to hongfeng-73@163.com.

## References

[1] Wang L Wang K Liu LJ Zhang YY Shu HN Wang K. Associations of daytime napping with incident cardiovascular diseases and hypertension in Chinese adults: A nationwide cohort study. Biomed Environ Sci. (2022) 35:22–34. doi: 10.3967/bes2022.004 35078559

[2] Wannamethee SG. Napping and Obesity in Adults – What do we Know? Curr Diabetes Rep. (2024) 24:237–43. doi: 10.1007/s11892-024-01551-5 PMC11405488 39145893

[3] Li H Shi Z Chen X Wang J Ding J Geng S. Relationship between obesity indicators and hypertension–diabetes comorbidity in an elderly population: a retrospective cohort study. BMC Geriatrics. (2023) 23:789. doi: 10.1186/s12877-023-04510-z 38036950 PMC10691080

[4] Kitamura K Watanabe Y Nakamura K Takano C Hayashi N Sato H. Short daytime napping reduces the risk of cognitive decline in community-dwelling older adults: a 5-year longitudinal study. BMC Geriatr. (2021) 21:474. doi: 10.1186/s12877-021-02418-0 34454431 PMC8401113

[5] Zhang W Zhou B Jiang C Jin Y Zhu T Zhu F. Associations of daytime napping and nighttime sleep quality with depressive symptoms in older Chinese: the Guangzhou biobank cohort study. BMC Geriatr. (2023) 23:875. doi: 10.1186/s12877-023-04579-6 38114908 PMC10731710

[6] Leng Y Wainwright NWJ Cappuccio FP Surtees PG Hayat S Luben R. Daytime napping and the risk of all-cause and cause-specific mortality: A 13-year follow-up of a British population. Am J Epidemiol. (2014) 179:1115–24. doi: 10.1093/aje/kwu036 PMC3992821 24685532

[7] Han B Chen WZ Li YC Chen J Zeng ZQ. Sleep and hypertension. Sleep Breath. (2020) 24:351–6. doi: 10.1007/s11325-019-01907-2 PMC7127991 31402441

[8] Wang H Chen L Shen D Cao Y Zhang X Xie K. Association of daytime napping in relation to risk of diabetes: evidence from a prospective study in Zhejiang, China. Nutr Metab (Lond). (2021) 18:18. doi: 10.1186/s12986-021-00545-4 33557863 PMC7869458

[9] He J Ouyang F Qiu D Duan Y Luo D Xiao S. Association of nap duration after lunch with prevalence of metabolic syndrome in a Chinese government employee population. Int J Environ Res Public Health. (2020) 17:4268. doi: 10.3390/ijerph17124268 32549270 PMC7344757

[10] Danaei G Finucane MM Lu Y Singh GM Cowan MJ Paciorek CJ. National, regional, and global trends in fasting plasma glucose and diabetes prevalence since 1980: systematic analysis of health examination surveys and epidemiological studies with 370 country-years and 2·7 million participants. Lancet. (2011) 378:31–40. doi: 10.1016/S0140-6736(11)60679-X 21705069

[11] Tu Q Hyun K Lin S Hafiz N Manandi D Li E. Impacts of hypertension and diabetes on the incidence of cardiovascular diseases and all-cause mortality: findings from the China Health and Retirement Longitudinal Study cohort. J Hypertens. (2025) 43:623–30. doi: 10.1097/HJH.0000000000003946 39791435

[12] Liu H Feng Z Zhang W Liu Y Xiong N Chen W. Prevalence of cognitive impairment and its associated factors in type 2 diabetes mellitus patients with hypertension in Hunan, China: a cross-sectional study. Front Psychiatry. (2024) 15:1445323. doi: 10.3389/fpsyt.2024.1445323 39748907 PMC11693732

[13] Hu Y Wang Z He H Pan L Tu J Shan G. Prevalence and patterns of multimorbidity in China during 2002-2022: A systematic review and meta-analysis. Ageing Res Rev. (2024) 93:102165. doi: 10.1016/j.arr.2023.102165 38096988

[14] Du Y Liu L Zhao Y Huang J Golden AR Cai L. Ethnic disparities in prevalence of chronic non-communicable diseases and its multimorbidity among older adults in rural southwest China. BMC Public Health. (2023) 23:1217. doi: 10.1186/s12889-023-16161-1 37353785 PMC10288692

[15] Qiu L Wang W Sa R Liu F. Prevalence and risk factors of hypertension, diabetes, and dyslipidemia among adults in northwest China. Int J Hypertension. (2021) 2021:5528007. doi: 10.1155/2021/5528007 PMC8055385 33936811

[16] Lin W-Q Yuan L-X Sun M-Y Wang C Liang E-M Li Y-H. Prevalence and patterns of multimorbidity in chronic diseases in Guangzhou, China: a data mining study in the residents’ health records system among 31–708 community-dwelling elderly people. BMJ Open. (2022) 12:e056135. doi: 10.1136/bmjopen-2021-056135 PMC9134174 35613781

[17] Tian M Feng D Chen X Chen Y Sun X Xiang Y. China’s rural public health system performance: a cross-sectional study. PloS One. (2013) 8:e83822. doi: 10.1371/journal.pone.0083822 24386284 PMC3873379

[18] Hu Y Zhang Y Zhong J Wang Y Zhou E Hong F. Association between obesity phenotypes and dietary patterns: A two-step cluster analysis based on the China multi-ethnic cohort study. Preventive Med. (2024) 187:108100. doi: 10.1016/j.ypmed.2024.108100 39146982

[19] Hong C Wu C Ma P Cui H Chen L Li R. Positive association of nap duration with risk of non-alcoholic fatty liver disease in an occupational population in Guangdong Province, China: a cross-sectional study. BMC Gastroenterol. (2022) 22:185. doi: 10.1186/s12876-022-02246-5 35413791 PMC9004137

[20] Gao Y Tang W Mao D Chen L Ding X. Association between Nocturnal Sleep Duration and Insomnia symptoms with depressive symptoms among 44,900 Chinese Han adults aged 30–79 in Southwest China. BMC Psychiatry. (2023) 23:127. doi: 10.1186/s12888-023-04601-6 36849922 PMC9972728

[21] Hu P Vinturache A Chen Y Ding G Zhang Y. Joint association of sleep onset time and sleep duration with cardiometabolic health outcome. J Am Heart Assoc. (2024) 13:e034165. doi: 10.1161/JAHA.123.034165 38874059 PMC11255762

[22] Hou Z Chen Y Sun Y Song C Deng H Cheng N. Sleep duration and insomnia with comorbid depression and anxiety symptoms in Chinese adults: A cross-sectional study. NSS. (2023) 15:1079–91. doi: 10.2147/NSS.S440584 PMC10749553 38146513

[23] Song Y Liu H Gu K Liu Y. U-shaped association between sleep duration and frailty in Chinese older adults: a cross-sectional study. Front Public Health. (2025) 12:1464734. doi: 10.3389/fpubh.2024.1464734 39839383 PMC11746093

[24] Makino S Al-Abri MA. Editorial: Effects of midday naps on glycemic control of diabetic patients. Front Endocrinol. (2024) 15:1430924. doi: 10.3389/fendo.2024.1430924 PMC11148864 38836219

[25] Fu J Zhang X Moore JB Wang B Li R. Midday nap duration and hypertension among middle-aged and older Chinese adults: A nationwide retrospective cohort study. Int J Environ Res Public Health. (2021) 18:3680. doi: 10.3390/ijerph18073680 33916042 PMC8037516

[26] Marcos-Delgado A Martín-Sánchez V Martínez-González MÁ Corella D Salas-Salvadó J Schröder H. Objectively measured sleep duration and health-related quality of life in older adults with metabolic syndrome: A one-year longitudinal analysis of the PREDIMED-plus cohort. Nutrients. (2024) 16:2631. doi: 10.3390/nu16162631 39203769 PMC11357069

[27] Liu W Wu Q Wang M Wang P Shen N. Prospective association between sleep duration and cognitive impairment: Findings from the China Health and Retirement Longitudinal Study (CHARLS). Front Med. (2022) 9:971510. doi: 10.3389/fmed.2022.971510 PMC9485441 36148464

[28] Feinberg I Maloney T March JD. Precise conservation of NREM period 1 (NREMP1) delta across naps and nocturnal sleep: implications for REM latency and NREM/REM alternation. Sleep. (1992) 15:400–3. doi: 10.1093/sleep/15.5.400 1455122

[29] Hu P Vinturache A Chen Y Ding G Zhang Y. Joint association of sleep onset time and sleep duration with cardiometabolic health outcome. J Am Heart Assoc. (2024). doi: 10.1161/JAHA.123.034165 PMC11255762 38874059

[30] Kahn SE. The relative contributions of insulin resistance and beta-cell dysfunction to the pathophysiology of Type 2 diabetes. Diabetologia. (2003) 46:3–19. doi: 10.1007/s00125-002-1009-0 12637977

[31] Brosolo G Da Porto A Bulfone L Vacca A Bertin N Scandolin L. Insulin resistance and high blood pressure: mechanistic insight on the role of the kidney. Biomedicines. (2022) 10:2374. doi: 10.3390/biomedicines10102374 36289636 PMC9598512

[32] Ertuglu LA Elijovich F Laffer CL Kirabo A. Salt-sensitivity of blood pressure and insulin resistance. Front Physiol. (2021) 12:793924. doi: 10.3389/fphys.2021.793924 34966295 PMC8711096

[33] Sakr HF Sirasanagandla SR Das S Bima AI Elsamanoudy AZ. Insulin resistance and hypertension: mechanisms involved and modifying factors for effective glucose control. Biomedicines. (2023) 11:2271. doi: 10.3390/biomedicines11082271 37626767 PMC10452601

[34] Leong RLF Yu N Ong JL Ng ASC Jamaluddin SA Cousins JN. Memory performance following napping in habitual and non-habitual nappers. SLEEP. (2021) 44:zsaa277. doi: 10.1093/sleep/zsaa277 33313925 PMC8193563

[35] Souabni M Souabni MJ Hammouda O Romdhani M Trabelsi K Ammar A. Benefits and risks of napping in older adults: A systematic review. Front Aging Neurosci. (2022) 14:1000707. doi: 10.3389/fnagi.2022.1000707 36337699 PMC9634571

[36] Wang Z Chen Y Tang S Chen S Gong S Jiang X. Dietary diversity and nutrient intake of han and dongxiang smallholder farmers in poverty areas of northwest China. Nutrients. (2021) 13:3908. doi: 10.3390/nu13113908 34836163 PMC8621596

[37] Cai L Wang X-M Fan L-M Cui W-L Golden AR. Socioeconomic disparities in prevalence and behaviors of smoking in rural Southwest China. BMC Public Health. (2019) 19:1117. doi: 10.1186/s12889-019-7455-0 31412820 PMC6694669

[38] Vu THL Bui TTQ Tran QB Pham QN Lai DT Le TH. Comorbidities of diabetes and hypertension in Vietnam: current burden, trends over time, and correlated factors. BMC Public Health. (2023) 23:2419. doi: 10.1186/s12889-023-17383-z 38053119 PMC10696748

[39] Xin C Zhang B Fang S Zhou J. Daytime napping and successful aging among older adults in China: a cross-sectional study. BMC Geriatrics. (2020) 20:2. doi: 10.1186/s12877-019-1408-4 31898552 PMC6941277

[40] Jung K-I Song C-H Ancoli-Israel S Barrett-Connor E. Gender differences in nighttime sleep and daytime napping as predictors of mortality in older adults: The Rancho Bernardo Study. Sleep Med. (2013) 14:12–9. doi: 10.1016/j.sleep.2012.06.004 PMC3542414 22951185

[41] Jiao J Feng X Gong A Yao Y. Association between reproductive lifespan and multimorbidity among Chinese postmenopausal women. Menopause. (2024) 31:945–51. doi: 10.1097/GME.0000000000002419 39078652

[42] Mackie P Weerasekara I Crowfoot G Janssen H Holliday E Dunstan D. What is the effect of interrupting prolonged sitting with frequent bouts of physical activity or standing on first or recurrent stroke risk factors? A scoping review. PloS One. (2019) 14:e0217981. doi: 10.1371/journal.pone.0217981 31194799 PMC6563984

[43] Fritschi C Quinn L. Fatigue in patients with diabetes: A review. J Psychosomatic Res. (2010) 69:33–41. doi: 10.1016/j.jpsychores.2010.01.021 PMC2905388 20630261

[44] Meyer S. How adrenal fatigue from chronic stress affects your health. Adv Obes Weight Manag Control (2016) 5:248. doi: 10.15406/aowmc.2016.05.00129

[45] Quadra MR Santos LPD Schäfer AA Meller F de O. Influence of sleep and chrononutrition on hypertension and diabetes: a population-based study. Cad Saude Publica. (2022) 38:e00291021. doi: 10.1590/0102-311XPT291021 35894369

[46] Valenzuela PL Santos-Lozano A Castillo-García A Lucia A. Diabetes and hypertension: physical activity and body mass index matter: insights from half a million people. Eur J Prev Cardiol. (2022) 29:1710–3. doi: 10.1093/eurjpc/zwac053 35403194

[47] Qin B Li Z Xia G Wang X Bai R. Bidirectional relationship between afternoon naps and depressive symptoms in Chinese middle-aged and older adults: Evidence from a nationally representative cohort study. J Affect Disord. (2025) 375:380–9. doi: 10.1016/j.jad.2025.01.122 39889928

