# Supplementary material for: Nap duration and its association with hypertension-diabetes comorbidity in minority populations: evidence from the CMEC study
Source: Front Endocrinol (Lausanne). 2025 Apr 28;16:1563944. doi: 10.3389/fendo.2025.1563944 (PMC12066342; doi:10.3389/fendo.2025.1563944)
Supplement: Supplementary file 1 [file Table1.docx]

**Table S1.** Association of nap duration and HDC in multivariable logistic regression when stratified by sex

| **Nap duration** | **N** | **Model 1^a^** |  | **Model 2^b^** |  | **Model 3^c^** |  |
| --- | --- | --- | --- | --- | --- | --- | --- |
|  |  | ***OR* (95% CI)** | ***P* value** | ***OR* (95% CI)** | ***P* value** | ***OR* (95% CI)** | ***P* value** |
| **Female** |  |  |  |  |  |  |  |
| 0h | 6801 | Ref |  | Ref |  | Ref |  |
| [0h, 0.5h) | 1917 | 0.917 (0.646, 1.275) | 0.618 | 1.165 (0.816, 1.631) | 0.385 | 1.098 (0.758, 1.557) | 0.610 |
| [0.5h, 1h) | 2367 | 1.206 (0.903, 1.593) | 0.196 | **1.346 (1.003, 1.788)** | 0.043 | 1.287 (0.952, 1.722) | 0.094 |
| >1h | 1167 | **1.504 (1.056, 2.096)** | 0.019 | **1.745 (1.219, 2.450)** | 0.002 | **1.677 (1.165, 2.369)** | 0.004 |
| **Male** |  |  |  |  |  |  |  |
| 0h | 2955 | Ref |  | Ref |  | Ref |  |
| [0h, 0.5h) | 1045 | **1.689 (1.243, 2.279)** | <0.001 | **1.346 (1.013, 1.781)** | 0.039 | **1.749 (1.270, 2.387)** | <0.001 |
| [0.5h, 1h) | 1593 | **1.935 (1.417, 2.624)** | <0.001 | **1.439 (1.081, 1.909)** | 0.012 | **1.754 (1.269, 2.402)** | <0.001 |
| >1h | 896 | **1.428 (1.027, 1.972)** | 0.032 | **1.753 (1.269, 2.404)** | <0.001 | **1.649 (1.174, 2.296)** | 0.003 |

^a^ Covariables in model 1: no confounding factors were adjusted. ^b^ Covariables in model 2: age, ethnicity, ^c^ Covariables in model 3: Model 2 + residence, annual household income, central obesity, smoke, drink, salt intake, physical activity, sleep duration, sleep disorder. OR indicates odds ratio; Ref, reference; CI, confidence interval.

**Table S2.** Association of nap duration and HDC in multivariable logistic regression when stratified by age

| **Nap duration** | **N** | **Model 1^a^** |  | **Model 2^b^** |  | **Model 3^c^** |  |
| --- | --- | --- | --- | --- | --- | --- | --- |
|  |  | ***OR* (95% CI)** | ***P* value** | ***OR* (95% CI)** | ***P* value** | ***OR* (95% CI)** | ***P* value** |
| **<45** |  |  |  |  |  |  |  |
| 0h | 2437 | Ref |  | Ref |  | Ref |  |
| [0h, 0.5h) | 995 | 1.230 (0.465, 2.967) | 0.655 | 1.123 (0.423, 2.721) | 0.803 | 0.981 (0.358, 2.464) | 0.968 |
| [0.5h, 1h) | 1164 | 1.773 (0.803, 3.854) | 0.147 | 1.535 (0.692, 3.354) | 0.28 | 1.440 (0.616, 3.287) | 0.388 |
| >1h | 526 | 1.988 (0.701, 4.983) | 0.161 | 1.853 (0.650, 4.678) | 0.2117 | 1.722 (0.529, 4.820) | 0.324 |
| **45-60** |  |  |  |  |  |  |  |
| 0h | 4443 | Ref |  | Ref |  | Ref |  |
| [0h, 0.5h) | 1454 | **1.465 (1.070, 1.986)** | 0.015 | 1.354 (0.986, 1.838) | 0.056 | 1.171 (0.834, 1.625) | 0.353 |
| [0.5h, 1h) | 1800 | **1.444 (1.078, 1.922)** | 0.012 | 1.303 (0.970, 1.739) | 0.075 | 1.175 (0.864, 1.587) | 0.299 |
| >1h | 919 | **2.241 (1.623, 3.060)** | <0.001 | **2.096 (1.511, 2.875)** | <0.001 | **2.106 (1.499, 2.928)** | <0.001 |
| **>60** |  |  |  |  |  |  |  |
| 0h | 2876 | Ref |  | Ref |  | Ref |  |
| [0h, 0.5h) | 513 | **1.843 (1.285, 2.596)** | <0.001 | **1.793 (1.249, 2.530)** | 0.001 | **1.557 (1.067, 2.234)** | 0.019 |
| [0.5h, 1h) | 996 | **1.439 (1.066, 1.926)** | 0.016 | **1.404 (1.037, 1.885)** | 0.026 | 1.336 (0.976, 1.813) | 0.066 |
| >1h | 618 | 1.338 (0.923, 1.900) | 0.112 | 1.311 (0.900, 1.872) | 0.147 | 1.241 (0.842, 1.794) | 0.262 |

^a^ Covariables in model 1: no confounding factors were adjusted. ^b^ Covariables in model 2: sex, ethnicity, ^c^ Covariables in model 3: Model 2 + residence, annual household income, central obesity, smoke, drink, salt intake, physical activity, sleep duration, sleep disorder. OR indicates odds ratio; Ref, reference; CI, confidence interval.

**Table S3.** Association of nap duration and HDC in multivariable logistic regression when stratified by ethnicity

| **Nap duration** | **N** | **Model 1^a^** |  | **Model 2^b^** |  | **Model 3^c^** |  |
| --- | --- | --- | --- | --- | --- | --- | --- |
|  |  | ***OR* (95% CI)** | ***P* value** | ***OR* (95% CI)** | ***P* value** | ***OR* (95% CI)** | ***P* value** |
| **Dong** |  |  |  |  |  |  |  |
| 0h | 3709 | Ref |  | Ref |  | Ref |  |
| [0h, 0.5h) | 1021 | **1.623 (1.111, 2.333)** | 0.01 | **1.907 (1.295, 2.766)** | <0.001 | **1.535 (1.016, 2.283)** | 0.038 |
| [0.5h, 1h) | 1519 | 1.237 (0.866, 1.746) | 0.233 | 1.268 (0.883, 1.799) | 0.19 | 1.130 (0.774, 1.630) | 0.519 |
| >1h | 981 | **1.812 (1.255, 2.580)** | 0.001 | **1.577 (1.084, 2.261)** | 0.015 | **1.599 (1.082, 2.333)** | 0.016 |
| **Miao** |  |  |  |  |  |  |  |
| 0h | 3172 | Ref |  | Ref |  | Ref |  |
| [0h, 0.5h) | 848 | 1.183 (0.777, 1.756) | 0.417 | 1.396 (0.909, 2.090) | 0.115 | 1.102 (0.696, 1.699) | 0.669 |
| [0.5h, 1h) | 1068 | **1.455 (1.018, 2.054)** | 0.036 | **1.535 (1.065, 2.185)** | 0.019 | 1.284 (0.876, 1.860) | 0.192 |
| >1h | 461 | **2.216 (1.438, 3.319)** | <0.001 | **2.179 (1.402, 3.303)** | <0.001 | **1.920 (1.208, 2.975)** | 0.004 |
| **Bouyei** |  |  |  |  |  |  |  |
| 0h | 2875 | Ref |  | Ref |  | Ref |  |
| [0h, 0.5h) | 1093 | 1.167 (0.792, 1.691) | 0.423 | 1.358 (0.915,1.981) | 0.119 | 1.251 (0.835, 1.846) | 0.267 |
| [0.5h, 1h) | 1373 | **1.403 (1.004, 1.947)** | 0.045 | 1.362 (0.970,1.900) | 0.071 | 1.392 (0.984, 1.957) | 0.059 |
| >1h | 621 | 1.494 (0.959, 2.261) | 0.066 | 1.466 (0.935, 2.234) | 0.084 | 1.406 (0.884, 2.174) | 0.136 |

^a^ Covariables in model 1: no confounding factors were adjusted. ^b^ Covariables in model 2: sex, age, ^c^ Covariables in model 3: Model 2 + residence, annual household income, central obesity, smoke, drink, salt intake, physical activity, sleep duration, sleep disorder. OR indicates odds ratio; Ref, reference; CI, confidence interval.
